# FOLFOX-HAIC combined with targeted immunotherapy for initially unresectable hepatocellular carcinoma: a real-world study

**DOI:** 10.3389/fimmu.2024.1471017

**Published:** 2024-11-26

**Authors:** Yan-Cen Lu, Yu-Chen Yang, Di Ma, Jun-qing Wang, Feng-Jie Hao, Xu-xiao Chen, Yong-jun Chen

**Affiliations:** Department of General Surgery, Hepatobiliary Surgery, Shanghai Institute of Digestive Surgery, Ruijin Hospital, Shanghai Jiaotong University School of Medicine, Shanghai, China

**Keywords:** hepatocellular carcinoma, targeted therapy, immunotherapy, combined therapy, conversion therapy, FOLFOX-HAIC, unresectable advanced cancer

## Abstract

**Background:**

Hepatic arterial infusion chemotherapy (HAIC) with the FOLFOX regimen has demonstrated efficacy in patients with unresectable hepatocellular carcinoma (HCC). The combined targeted and immunotherapy has emerged as a first-line treatment for liver cancer. In this study, we investigated the clinical efficacy and safety of FOLFOX-HAIC in combination with targeted immunotherapy in patients with untreated, unresectable HCC.

**Materials and methods:**

Data were collected from patients with initially unresectable HCC treated at Ruijin Hospital, affiliated with Shanghai Jiao Tong University School of Medicine, from June 2022 to June 2023. Tumor response and survival outcomes were assessed following the FOLFOX-HAIC combined with targeted immunotherapy, The safety was also evaluated through the incidence of related adverse events.

**Results:**

A total of 51 eligible patients were recruited. The objective response rate (ORR) based on mRECIST and RECIST 1.1 criteria were 60.8% and 45.1%, respectively. The surgical conversion rate was 25.5%. The median progression-free survival (PFS) was 15.2 months. The 1-year overall survival rate was 88.2%. Adverse events were observed in 98% patients, with 23.5% experiencing grade 3 or 4 adverse events.

**Conclusion:**

The FOLFOX-HAIC combined with targeted immunotherapy regimen is effective in patients with unresectable HCC, demonstrated by a high surgical conversion rate and manageable adverse effects. This regimen represents a potential novel first-line treatment option for HCC.

## Introduction

Liver cancer is among the most malignant gastrointestinal tumors, including hepatocellular carcinoma (HCC), cholangiocarcinoma, and mixed-cell carcinoma, with HCC being the fourth leading cause of cancer-related deaths worldwide. The etiology of liver cancer is diverse, with common causes including chronic viral hepatitis (such as hepatitis B and C virus infections), alcohol-related liver disease, and non-alcoholic fatty liver disease (1–3). Many patients are diagnosed at an advanced or late stage of unresectable liver cancer, often resulting in a poor prognosis. To convert unresectable liver cancer to resectable liver cancer, it is crucial to identify effective treatment methods to improve the survival time of patients with advanced liver cancer.

Hepatic arterial infusion chemotherapy (HAIC) is recommended in Asia as an alternative option for patients with unresectable HCC (4–6). The Japanese Society of Hepatology’s HCC practice guidelines have recognized HAIC as an effective treatment for locally advanced HCC. HAIC is a liver-directed therapy that delivers high concentrations of chemotherapeutic agents directly to liver tumors via the hepatic artery, achieving a strong tumor response while minimizing excessive exposure of normal liver parenchyma to chemotherapy drugs. This approach effectively reduces tumor burden with relatively low systemic toxicity (7, 8). The combination of targeted drugs and immune checkpoint inhibitors is a first-line treatment strategy for advanced liver cancer. Targeted drugs inhibit the growth, division, and spread of tumor cells by binding to specific molecular targets associated with the tumor (9). Immunotherapy drugs enhance the recognition and elimination of tumor cells by targeting immune cells (10). Compared to single-agent therapies, combination treatments have shown significant efficacy, markedly improving patient survival. For instance, the median overall survival (mOS) with the combination of atezolizumab and bevacizumab can reach 19.2 months, the median progression-free survival (mPFS) was 6.9 months, and the objective response rate (ORR) was 30% (11).

Combining the advantages of HAIC and targeted immunotherapy, the integration of these therapies as a systemic treatment approach is still under exploration. This retrospective study examines the efficacy and safety of HAIC combined with targeted immunotherapy drugs in the treatment of advanced unresectable liver cancer.

## Materials and methods

### Patient information

Clinical data were collected from patients with initially unresectable HCC treated at Ruijin Hospital, affiliated with Shanghai Jiao Tong University School of Medicine, from June 2022 to June 2023. The inclusion criteria were: (1) Diagnosis of primary hepatocellular carcinoma based on pathology or imaging; (2) Tumors occupying more than 50% of the liver volume or associated with significant liver cirrhosis, multiple bilobar lesions, or invasion of major vascular structures, making it impossible to ensure negative surgical margins or zero residual disease, thus assessed as initially unresectable; (3) At least one measurable target lesion based on the Response Evaluation Criteria in Solid Tumors (RECIST) and the modified RECIST (mRECIST); (4) Completion of at least two cycles of hepatic arterial infusion chemotherapy combined with targeted immunotherapy; (5) No prior anti-HCC treatment; (6) Child-Pugh class A/B liver function and Eastern Cooperative Oncology Group (ECOG) performance status (PS) score of 0-2; (7) No significant organ dysfunction in kidneys, heart, or brain. The exclusion criteria were: (1) Incomplete clinical data; (2) Previous HCC treatments; (3) Decompensated liver function or ECOG PS score of 3-4; (4) Severe dysfunction of the heart, kidneys, or other organs, or concurrent other malignancies; (5) Intolerance or allergy to the medications; (6) Patients with extrahepatic metastases; (7)Barcelona Clinic Liver Cancer (BCLC) stage D.

### Research methods

#### Treatment protocol

Within one week before combined treatment, patients underwent enhanced CT or MRI scans to assess imaging characteristics. Relevant laboratory and examination information, including complete blood count, biochemistry, coagulation, and tumor immunology, were recorded. Patients lay supine. After disinfection and draping in the bilateral inguinal area, the femoral artery pulse was palpated on both sides to choose an appropriate puncture site (usually on the right side) (12, 13). Using local anesthesia, the Seldinger technique was employed to puncture the femoral artery, and after successful puncture, a 4F vascular sheath was placed. A contrast catheter was inserted through the vascular sheath, and selective catheterization of the celiac trunk or superior mesenteric artery was performed. Angiography indicates no vascular anomalies within the abdominal cavity. If ectopic vessels supplying the tumor were present, after arterial embolization, a selective catheterization to the tumor-feeding artery was conducted. A microcatheter was left in the tumor-feeding artery, the Y-valve was properly fixed, the right lower limb was secured, and the patient was returned to the ward. Postoperatively in the ward. A regimen of fluorouracil combined with oxaliplatin (FOLFOX) was continuously infused through the catheter using an infusion pump: (1)Oxaliplatin: 85 mg/m² (adjusted to 130 mg/m² if the tumor diameter was greater than 10 cm) infused over 2 hours. Typically, 130 mg/m² was used for large, well-vascularized tumors, while 85 mg/m² was used for smaller, less vascularized tumors. Dosage adjustments were necessary if a large tumor shrinks. (2)Leucovorin (Calcium Folinate): 200 mg/m² infused over 2 hours. (3)Fluorouracil: 400 mg/m² infused within 15 minutes, followed by 2400 mg/m² infused over 46 hours. The next chemotherapy cycle was repeated 3 or 4 weeks later with re-catheterization. If toxicity was intolerable, treatment may be interrupted when grade 3 or 4 adverse events occurred.

### Targeted immunotherapy

After a single HAIC chemotherapy session, targeted drugs and immunotherapeutic agents were selected based on the patients’ preferences and their financial situations. Common targeted and immune combinations include: (1) Atezolizumab + Bevacizumab; (2) Sintilimab + Bevacizumab biosimilar; (3) Lenvatinib or Donafenib combined with Tislelizumab or Pembrolizumab or camrelizumab. Before or after the first HAIC session, patients were intravenously administered anti-PD-1 antibodies every 3 weeks (200-mg tislelizumab, 200-mg camrelizumab, or 200-mg pembrolizumab). For anti-angiogenesis treatment, the patients were administered 8-mg lenvatinib orally once daily, 200-mg Donafenib orally twice daily. Patients in the atezolizumab + bevacizumab group were intravenously administered atezolizumab (1200 mg) plus bevacizumab (15 mg/kg) or sintilimab (200 mg) plus bevacizumab biosimilar (IBI305) every 3 weeks. Generally, each treatment cycle lasted 3 weeks and was repeated. If severe adverse reactions occur during the combined medication period, dose reductions and appropriate extensions of the treatment cycle were considered.

### Evaluation of treatment effectiveness

After two HAIC sessions, enhanced CT or MRI re-examinations were conducted, and relevant indicators such as complete blood count, biochemistry, and tumor markers were collected. Tumor efficacy was assessed according to the RECIST 1.1 and the mRECIST criteria. The assessments were as follows:Complete Response(CR); Partial Response (PR); Stable Disease (SD); Progressive Disease (PD); The Overall Response Rate (ORR) was calculated as the sum of PR and CR. The Disease Control Rate (DCR) includes PR, CR, and SD. Additionally, the surgical conversion rate and Progression-Free Survival (PFS) were evaluated.

### Statistical analysis

Relevant data were analyzed using the statistical software SPSS 26.0. Measurement data following a normal distribution were expressed as mean ± standard deviation; data not following a normal distribution were expressed as median and interquartile range. Survival analysis was performed using Kaplan-Meier curves. The Cox proportional hazards regression model was used for univariate and multivariate analyses of risk factors affecting Progression-Free Survival (PFS). All statistical methods calculated the 95% confidence interval (95% CI), with a p-value < 0.05 defined as statistically significant.

## Results

### Clinical characteristics of patients

In this study, a total of 51 patients with initially unresectable liver cancer who met the criteria were included. The included patients were mostly middle-aged and elderly, predominantly male (92.2%). The mean age of the enrolled patients was 56.1 ± 10.7 years. Most patients had chronic hepatitis B virus infection (74.5%), with 46 patients (90.2%) having a background of liver cirrhosis. Upon evaluation, 40 patients (78.4%) had a maximum tumor diameter of ≥7 cm (the 7 cm threshold was used for grouping based on previous study (7) and subsequent statistical analyses, and Univariate and multivariate analyses revealed that hepatocellular carcinoma smaller than 7 cm may have a better prognosis after treatment). Additionally, 35 patients (68.6%) had portal vein tumor thrombus, and 27 patients (52.9%) had multiple tumors. All patients were in Child-Pugh grade A or B. Overall, the tumor stages were advanced with a high burden. Among them, 49 patients (96.1%) were in BCLC stages B and C. 13 patients underwent Hepatectomy for HCC after combined treatment (**Table 1**). A total of 151 HAIC treatments were administered, combined with molecular targeted and immunotherapy. The choice of targeted therapy and immunotherapy drugs depended on the patients’ preferences and their financial situations. Eight patients opted for lenvatinib combined with camrelizumab; two patients chose lenvatinib combined with pembrolizumab; three patients selected lenvatinib combined with tislelizumab; three patients chose donafenib combined with sintilimab; three opted for donafenib combined with pembrolizumab; seventeen patients selected atezolizumab combined with bevacizumab, and fifteen patients chose sintilimab combined with a bevacizumab biosimilar. All drugs were administered according to their standard dosages. The median interval between HAIC therapy was 26 days. The median interval between targeted immunotherapy was 23 days.

**Table 1:** Baseline clinical characteristics of the included patients (n = 51).

Characteristics	Patients
Gender	
Male	47 (92.2%)
Female	4 (7.8%)
**Age**	56.1 ± 10.7
ECOG grade	
0	32 (62.7%)
1	19 (37.3%)
Etiology	
HBV	38 (74.5%)
HCV	4 (7.8%)
Other reasons	9 (17.6%)
**Cirrhosis**	46 (90.2%)
Total bilirubin	
≥34umol/L	9 (17.6%)
<34umol/L	42 (82.4%)
**Hypoproteinemia**	22 (43.1%)
Child-Pugh	
A	29 (56.9%)
B	22 (43.1%)
PVTT 35 (68.6%)	
Vp 1	10 (28.6%)
Vp 2	17 (48.6%)
Vp 3	8 (22.8%)
Number of tumors	
Single	24 (47.1%)
Multiple	27 (52.9%)
Tumor diameter	
≥5cm	40 (78.4%)
<5cm	11 (21.6%)
AFP	
≥400ng/mL	28 (54.9%)
<400ng/mL	23 (45.1%)
BCLC	
A	2 (3.9%)
B	11 (21.6%)
C	38 (74.5%)
Surgical conversion	13 (25.5%)

HBV, hepatitis b virus; HCV, hepatitis c virus; PVTT, portal vein tumor thrombus; AFP, α-fetoprotein; BCLC, barcelona clinic liver cancer.

### Tumor response and patient survival

**Figure 1:**
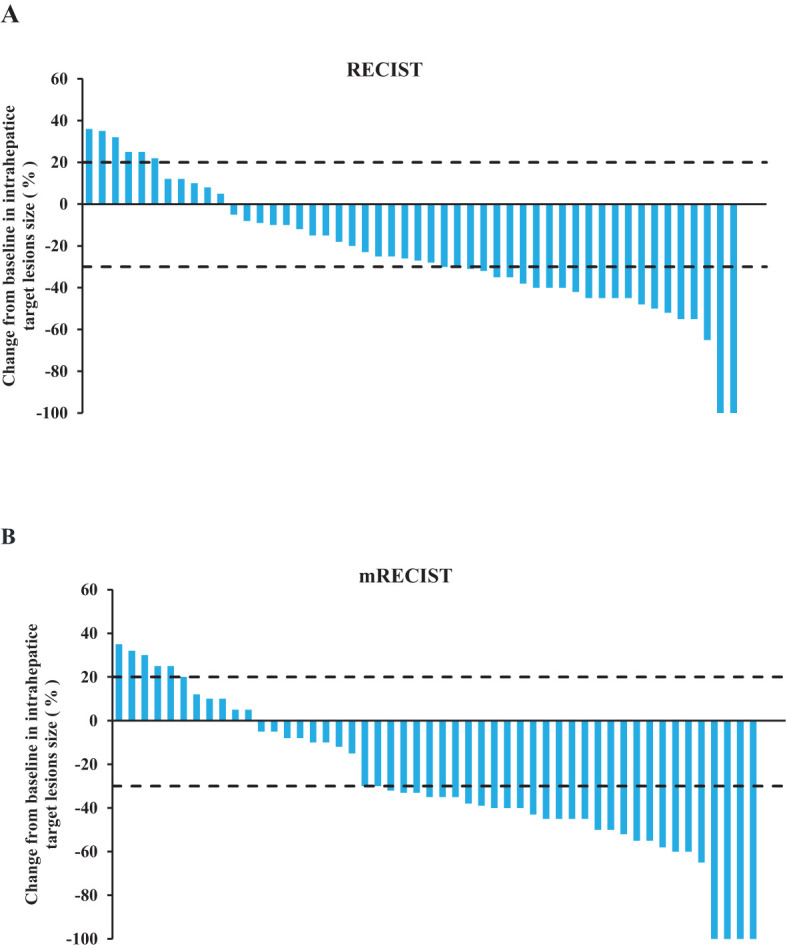
Tumor treatment response. **(A)** The best percentage change of the target lesion relative to the baseline according to RECIST v1.1. **(B)** The best percentage change of the target lesion relative to the baseline according to mRECIST.

According to RECIST v1.1 evaluation, the numbers of CR, PR, SD, and PD cases were 2 (3.9%), 21 (41.2%), 22 (43.1%), and 6 (11.8%), respectively. The ORR was 45.1% and the DCR was 88.2%. And according to mRECIST evaluation, the numbers of CR, PR, SD, and PD cases were 4 (7.8%), 27 (52.9%), 14 (27%), and 6 (11.8%), respectively. The ORR was 60.8% and the DCR was 88.2%. After combined treatment, 13 patients underwent liver cancer resection, with a surgical conversion rate of 25.5% (**Table 2**). The change in the intrahepatic target lesion size of the patients is shown in **Figure 1**.

**Table 2:** Tumor treatment response assessment.

Characteristics	RECIST	mRECIST
Complete response (CR)	2 (3.9%)	4 (7.8%)
Partial response (PR)	21 (41.2%)	27 (52.9%)
Stable disease (SD)	22 (43.1%)	14 (27.5%)
Progressive disease (PD)	6 (11.8%)	6 (11.8%)
Objective response rate (ORR)	23 (45.1%)	31 (60.8%)
Disease control rate (DCR)	45 (88.2%)	45 (88.2%)

RECIST, Response Evaluation Criteria in Solid Tumors; mRECIST, modified RECIST.

### Typical cases of pathological remission achieved after combination therapy

**Figure 2:**
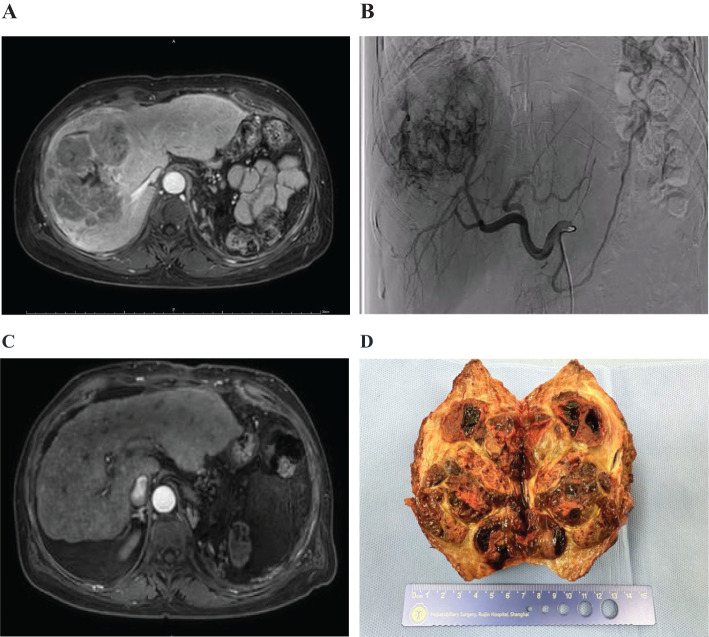
Imaging and pathological results of patients with hepatocellular carcinoma. **(A)** Multiple intrahepatic tumors were present. **(B)** Digital subtraction angiography (DSA) showed the tumors. **(C)** The lesions significantly reduced in size after treatment. **(D)** Postoperative gross pathological specimens were obtained.

A patient with multiple intrahepatic tumors(multiple lesions in the S5/S7/S8 segments of the liver), the largest being 13.9 cm in diameter (**Figure 2A**), and associated major vascular cancer thrombus, had a Child-Pugh B liver function and was at BCLC stage C for the tumors. After undergoing two treatments of FOLFOX-HAIC (**Figure 2B**) combined with sintilimab and a biosimilar of bevacizumab, a follow-up evaluation showed significant reduction in the intrahepatic tumors. After the first treatment, the tumor was evaluated to have shrunk to 13.2cm, and after the second combination therapy, it was reduced to 10.7cm (**Figure 2C**), and partial remission was achieved according to mRICIST assessment. The combined treatment’s side effects were tolerable. Subsequently, a complete surgical resection was performed, and postoperative pathology revealed no residual cancer tissue, indicating a complete pathological response to the treatment (**Figure 2D**).

**Figure 3:**
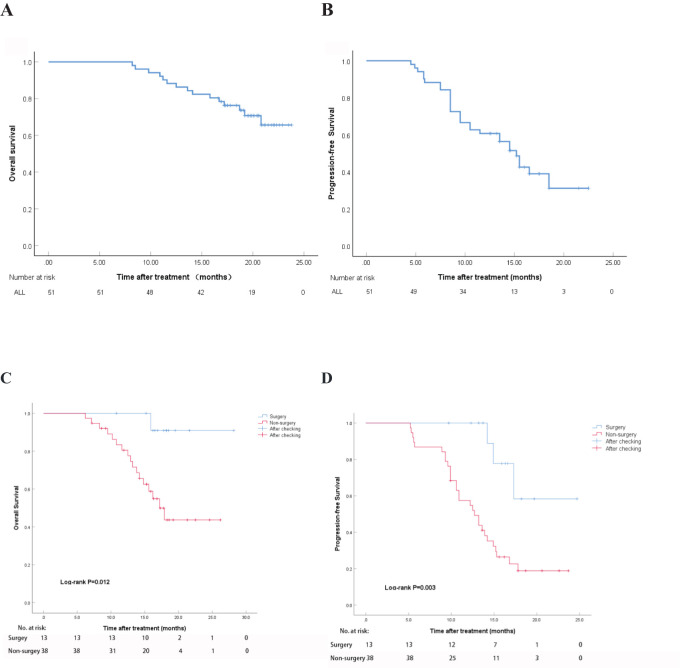
Overall survival time and progression-free survival period after treatment. **(A)** OS using mRECIST; **(B)** PFS using mRECIST; **(C)** OS in the surgery and non-surgery groups; **(D)** PFS in the surgery and non-surgery groups.

The median follow-up period was 16.6 months. According to the mRECIST criteria, the OS at 6, 12, 18 months were 100%, 88.2%, and 76.4%, respectively. The median OS (mOS) was not reached. The median follow-up interval was 3.6 months (**Figure 3A**). The PFS at 6 and 12 months were 88.2% and 60.9%, respectively, with a median PFS (mPFS) of 15.2 months (**Figure 3B**). After 18 months of follow-up, a total of 12 patients died, with a survival rate of 76.4%. In the subgroup analysis of the surgery and non-surgery groups, it was found that the mOS of the non-surgery group was 17.2 months (95% CI,14.4 to 20.0), and the mPFS was 12.7 months (95% CI,10.0 to 15.4). The mOS and mPFS of the surgical group were not achieved. Two subgroup analyses revealed that there was a statistically significant difference in OS (HR,0.12, P=0.012) and PFS (HR,0.20, P=0.003) between the surgery and non-surgery groups (**Figures 3C, D**).

Through univariate and multivariate COX analyses of PFS, it was found that age, portal vein tumor thrombus (PVTT), and tumor number were risk factors analyzed for their impact on prognosis. Specifically, it indicated that the older patients were more likely to experience disease progression compared to the younger ones. Patients with multiple tumor lesions were more likely to experience disease progression in contrast to the patients with a single tumor. Additionally, in the subgroup analysis based on PVTT, patients with PVTT had a potentially poor prognosis, who was more likely to experience disease recurrence (**Table 3**).

**Table 3:** Univariate and multivariate prognostic analysis.

Variable	Univariate Analysis		Multivariate Analysis	
	HR (95%CI)	*p*-value	HR (95%CI)	*p*-value
Gender (male/female)	1.018 (0.343~3.021)	0.974	
Age	1.038 (1.012~1.065)	0.004	1.030 (1.002~1.059)	0.034
ECOG grade (1/0)	1.280 (0.639~2.566)	0.487	
HBV (presence/absence)	1.418 (0.673~2.986)	0.359	
Cirrhosis (presence/absence)	1.167 (0.349~3.896)	0.802	
Hyperbilirubinemia (presence/absence)	0.630 (0.221~1.800)	0.389	
Hypoproteinemia (presence/absence)	0.939 (0.470~1.878)	0.859	
Tumor Number (multiple/single)	2.336 (1.126~4.848)	0.023	2.219 (1.067~4.617)	0.033
Tumor Diameter (≥10cm/<10cm)	1.302 (0.500~3.393)	0.589	
PVTT (presence/absence)	2.888 (1.244~6.705)	0.014	2.478 (1.051~5.842)	0.038
Child-Pugh (B/A)	1.786 (0.847~3.765)	0.128	
AFP (≥400ng/mL/<400ng/mL)	1.613 (0.800~3.251)	0.181	
BCLC (A/C)	1.877 (0.433~8.147)	0.400	
BCLC (B/C)	0.858 (0.350~2.103)	0.738	

HBV, hepatitis B virus; PVTT, portal vein tumor thrombus; AFP, alpha-fetoprotein; BCLC, barcelona clinic liver cancer.

### Adverse events

In this treatment study, adverse events related to a total of 151 HAIC treatments in 51 patients were statistically analyzed. All patients experienced adverse reactions of varying degrees (**Table 4**). The most common adverse reactions were elevated transaminases, thrombocytopenia, decreased albumin, abdominal pain, nausea, and vomiting. However, most patients experienced adverse reactions of grade 1-2, with no grade 5 adverse events observed. One patient (1.9%) experienced a grade 4 adverse reaction post-treatment, characterized by severe thrombocytopenia (41 x 10^9/L) accompanied by upper gastrointestinal bleeding symptoms, which improved after hemostasis via gastroscopy and platelet transfusion therapy. Twelve patients (23.5%) experienced severe post-chemotherapy reactions, including nausea, abdominal pain, vomiting, and difficulty eating, along with abnormal test indicators during repeated treatments. Chemotherapy drug perfusion was paused, and the patients were hospitalized for supportive symptomatic treatment with fluid supplementation, after which the symptoms improved, and subsequent drug perfusion chemotherapy was continued. The remaining 39 patients (74.5%) experienced grade 1-2 adverse reactions, mainly mild abdominal pain, vomiting, and elevated transaminases, which occurred during arterial infusion chemotherapy and improved with antispasmodic, analgesic, antiemetic, and liver-protective treatments. No catheter-related complications or treatment-related deaths occurred. The most common adverse reactions in patients who opted for FOLFOX-HAIC combined with lenvatinib and camrelizumab were vomiting, leukopenia, lypertension and Elevated transaminases. The most common adverse reactions in patients who selected FOLFOX-HAIC combined with atezolizumab combined with bevacizumab were anorexia, hypertension, elevated transaminases and elevated bilirubin. The most common adverse reactions in patients who chose FOLFOX-HAIC combined with sintilimab and a bevacizumab biosimilar were abdominal pain, anorexia, elevated transaminases and elevated bilirubin. Common adverse reactions in other patients include abdominal pain, anorexia, vomiting, elevated granulocytes, elevated transaminases and elevated bilirubin.

**Table 4:** Patient treatment-related adverse events.

Adverse Events	Grade 1	Grade 2	Grade 3	Grade 4
Abdominal pain	13 (25.40%)	5 (9.80%)	2 (3.90%)	0 (0.00%)
Anorexia	27 (52.90%)	7 (13.70%)	6 (11.80%)	1 (1.90%)
Vomiting	17 (33.30%)	3 (5.90%)	5 (9.80%)	1 (1.90%)
Fever	6 (11.80%)	1 (1.90%)	1 (1.90%)	0 (0.00%)
Diarrhea	9 (17.60%)	3 (5.90%)	3 (5.90%)	0 (0.00%)
Leukopenia	14 (27.50%)	6 (11.80%)	5 (9.80%)	0 (0.00%)
Thrombocytopenia	22 (43.10%)	12 (23.50%)	5 (9.80%)	1 (1.90%)
Elevated transaminases	28 (54.90%)	6 (11.80%)	5 (9.80%)	1 (1.90%)
Elevated bilirubin	16 (31.40%)	5 (9.80%)	2 (3.90%)	0 (0.00%)
Renal insufficiency	5 (9.80%)	0 (0.00%)	0 (0.00%)	0 (0.00%)
Hypoproteinemia	9 (17.60%)	6 (11.80%)	3 (5.90%)	0 (0.00%)
Cholangitis	0 (0.00%)	0 (0.00%)	0 (0.00%)	0 (0.00%)
Liver failure	0 (0.00%)	0 (0.00%)	0 (0.00%)	0 (0.00%)
Catheter dislodgement	0 (0.00%)	0 (0.00%)	0 (0.00%)	0 (0.00%)
Catheter thrombosis	0 (0.00%)	0 (0.00%)	0 (0.00%)	0 (0.00%)
Hypertension	12 (23.50%)	5 (9.80%)	2 (3.90%)	0 (0.00%)
Gastrointestinal bleeding	0 (0.00%)	0 (0.00%)	0 (0.00%)	1 (1.90%)

## Discussion

Hepatocellular carcinoma is one of the most common malignant tumors. Liver resection surgery is considered the preferred treatment method for curatively treating liver cancer, offering patients a chance for long-term survival. However, approximately 80% of HCC patients are ineligible for curative surgery at initial diagnosis due to tumor burden, tumor stage, or limited liver function reserve. The challenge of downstaging tumors to render initially unresectable HCC patients eligible for surgery and extending their survival has garnered increasing attention from researchers worldwide (14). Different treatment combinations, including local treatments such as interventional therapy and ablation, as well as systemic treatments combining targeted therapy and immunotherapy, are gaining prominence in conversion therapy aimed at tumor downstaging.

HAIC is based on the pathophysiological characteristics of most malignant liver tumors. The blood supply to hepatocellular carcinoma primarily comes from the hepatic artery, while the normal liver parenchyma is mainly supplied by the portal vein. HAIC achieves a high tumor response by delivering high concentrations of chemotherapeutic drugs directly to liver tumors through the hepatic artery, thus avoiding excessive exposure of the normal liver parenchyma to the drugs. Catheter arterial infusion chemotherapy involves continuous infusion of chemotherapeutic drugs into the hepatic artery via percutaneous puncture, increasing drug concentration, reducing systemic toxicity, and enhancing tumor uptake (15). The FOLFOX regimen was proposed by Chinese researchers, who found that this regimen had an ORR and DCR of 40.8% and 77.6%, respectively. The 6-month and 12-month survival rates were 71.4% and 55.1%. Additionally, in a clinical trial involving 262 liver cancer patients, it was discovered that compared to treatment with the single-target drug sorafenib, the FOLFOX regimen resulted in a higher OS (Overall Survival) (13.9 months vs. 8.2 months) (7, 8, 16, 17).

Currently, for patients with advanced unresectable liver cancer, systemic therapy with targeted drugs combined with immunotherapy is prioritized due to its proven efficacy (18). It is recommended to use either targeted therapy alone or in combination with Programmed Death Receptor-1 (PD-1/PD-L1) immune checkpoint inhibitors as the first-line treatment or alternative therapy for advanced HCC (19–23). In the IMbrave150 study, the combination of atezolizumab and bevacizumab showed superior OS and PFS compared to the single-agent sorafenib, providing better tumor efficacy and longer survival for patients. The ORR (Overall Response Rate) for the T+A group reached 30%, indicating the advantage of combining targeted therapy with immunotherapy over single-agent targeted therapy. The median OS in the global population was 19.2 months, and the combination achieved a 6% complete response rate. The ORIENT-32 study (24) results showed that, compared to the sorafenib group, the combination of sintilimab and a bevacizumab biosimilar reduced the risk of death by 43% and the risk of progression by 43%. The ORR in the combination therapy group was also significantly higher than in the sorafenib group. According to RECIST 1.1 criteria, the ORRs were 20.3% and 4.1% for the two groups, respectively; according to mRECIST criteria, the ORRs were 23.4% and 7.1%. The KeyNote 524 study (11) revealed that the combination of lenvatinib and pembrolizumab had an ORR of 41% and a DCR (Disease Control Rate) of 86%, demonstrating reliable anti-tumor activity. The BGB-A317-211 study found that the combination of tislelizumab and lenvatinib had an ORR of 41.9% and a DCR of 85.5%. Additionally, the SHR 310 study (25) discovered that the combination of camrelizumab and apatinib (C+R) significantly prolonged PFS and OS and improved the ORR.

The triple therapy regimen combining HAIC (Hepatic Arterial Infusion Chemotherapy) with targeted therapy and immunotherapy has also shown good safety and excellent efficacy (26, 27). Existing research results indicate that the HAIC + lenvatinib + PD-1 triple therapy group achieved longer PFS (11.1 months vs. 5.1 months, P<0.001) and overall survival (not reached vs. 11 months, P<0.001) compared to the standard single targeted drug lenvatinib group. It also showed a higher DCR (90.1% vs. 72.1%, P=0.005) and objective response rate [RECIST 1.1 criteria: 59.2% vs. 9.3%, P<0.001; new criteria for evaluating solid tumors (mRECIST criteria): 67.6% vs. 16.3%, P<0.001]. Additionally, 14.1% of patients in the triple therapy group achieved CR according to the mRECIST criteria (28).

In this study, we retrospectively included 51 patients with advanced unresectable liver cancer. After receiving combined FOLFOX-HAIC targeted and immune therapy, the median mPFS was 15.2 months, and mOS had not been reached. According to the mRECIST criteria, the ORR was 60.8%, and the DCR was 88.2%. Thirteen patients (25.5%) successfully obtained the opportunity for surgery after combined therapy, among which 4 patients achieved CR. A previous retrospective study (29) of HAIC combined with targeted immune therapy indicated that among 25 patients, 15 (60.0%) achieved conversion to surgical standards; 1 person refused surgery, and the remaining 14 underwent surgical resection. Among these, 7 patients (28.0%) achieved pathological CR after resection, with a recurrence-free survival of 13.17 months. In our study, the surgical conversion rate did not reach the expected results mentioned above. This may be due to a significant portion of the enrolled patients being classified as BCLC stage C, with high tumor burden and most having PVTT (portal vein tumor thrombosis). According to Zhang et al., (30) the combination of HAIC targeted immunotherapy demonstrated a significant ORR of 54.1%, DCR of 94.6%, a Surgical conversion rate of 29.6%. Common adverse effects include pain, fatigue and abnormal liver function. These results were similar to the results in this study. Lin (31) reported that when HAIC was combined with targeted immunotherapy, it achieved an ORR of 60.4%, along with a DCR of 100% based on mRECIST criteria. The median PFS (mPFS) was 13.9 months. Adverse events of different degrees were observed in all patients after receiving the combination therapy. Abdominal pain, nausea, vomiting, increased levels of transaminases, anorexia, fatigue, and diarrhea mainly occurred in the treatment. All patients recovered within a few days. Zhang (27) reported that the combination of HAIC, camrelizumab, and apatinib demonstrated an ORR of 77.1%and a DCR of 97.1% in treating advanced HCC patients. The median PFS (mPFS) was recorded at 10.38 months which was shorter than the mPFS in this study. This year’s global multicenter clinical study EMERALD-1 (32) revealed the effectiveness and safety of TACE combined with targeted immunotherapy. We compared this study with the EMERALD-1 study and found that according to the up-to-7 criteria, patients in this study had higher tumor burden levels, with more patients exceeding the up-to-7 criteria (90.2% vs. 52.5%, p<0.001), while the objective response rate and disease control rate in this study were higher (ORR: 60.8% vs. 43.6%, p=0.024; DCR: 88.2% vs. 65.8%, p<0.001).

Adverse reactions caused by chemotherapeutic drugs in HAIC are relatively common, but most are mild (grades 1-2). Studies (33, 34) have found that adverse reactions to HAIC alone occur at a rate of 88.1%, with leukopenia (9%) being the most common. In this study, the total number of adverse reactions after HAIC combined with targeted immunotherapy was 46 (90.2%). Among grade 1-2 adverse reactions, thrombocytopenia and elevated transaminases were the most common, with anorexia and vomiting being the predominant reactions after chemotherapy. These patients showed improvement after conservative treatment with liver protection, antiemetics, and gastric protection, and tolerated subsequent treatments. Among grade 3 adverse reactions, anorexia, leukopenia, thrombocytopenia, and elevated transaminases were more common, and these patients were also able to maintain subsequent treatment after receiving appropriate interventions.

This study has several limitations. First, it is a retrospective, single-center study; hence, it is inevitably subject to unknown selection biases, which reduce the generalizability of the findings. Second, the small sample size and the lack of a control group diminish the reliability of the evidence and increase the comparative error. Therefore, further research with a control group in a larger population is needed. Finally, the relatively short follow-up period may reduce the quality of the observed effectiveness and affect the estimates of ORR, PFS and OS.

## Conclusion

The FOLFOX-HAIC combined with targeted immunotherapy regimen is effective in patients with unresectable HCC, demonstrated by a high surgical conversion rate and manageable adverse effects. This regimen represents a potential novel first-line treatment option for HCC.

## Data Availability

The raw data supporting the conclusions of this article will be made available by the authors, without undue reservation.
