# Supplementary material for: FOLFOX-HAIC combined with targeted immunotherapy for initially unresectable hepatocellular carcinoma: a real-world study
Source: Front Immunol. 2024 Nov 26;15:1471017. doi: 10.3389/fimmu.2024.1471017 (PMC11628521; doi:10.3389/fimmu.2024.1471017)
Supplement: Supplementary file 1 [file DataSheet1.pdf]

7. the similarities and differences between this study and previous published studies

Title/Year	The similarities	The differences
**THIS RESEARCH**<br>FOLFOX-HAIC Combined with Targeted Immunotherapy for Initially Unresectable Hepatocellular Carcinoma: A Real-World Study/2024		
Hepatic arterial infusion chemotherapy combined with anti-PD-1/PD-L1 immunotherapy and molecularly targeted agents for advanced hepatocellular carcinoma: a real world study/2023	1. single-center retrospective studies<br>2. analysis of the efficacy and safety of FOLFOX-HAIC combined with Targeted Immunotherapy therapy for unresectable liver cancer<br>3. the diversification of treatment regimens<br>4. The objective response rates are similar.	1. Patients with extrahepatic metastases were also included in this study.<br>2. There are patients who have received only one combination therapy.
Efficacy and safety of targeted therapy plus immunotherapy combined with hepatic artery infusion chemotherapy (FOLFOX) for unresectable hepatocarcinoma/2024	1. single-center retrospective studies<br>2. analysis of the efficacy and safety of FOLFOX-HAIC combined with Targeted Immunotherapy therapy for unresectable liver cancer<br>3. the diversification of treatment regimens<br>4. The disease control rates are similar.	1. The sample size was larger, but the objective response rate was slightly lower.<br>2. There are patients who have received only one combination therapy.

8. the information of the 13 HCC patients who underwent resection

7 patients used the treatment of FOLFOX-HAIC combined with atezolizumab and bevacizumab

**Characteristics**	**Patients**
**Gender**	
Male	6 (85.7%)
Female	1 (14.3%)
**ECOG grade**	

0	4 (57.1%)
1	3 (42.9%)
**Etiology**	
HBV	5 (71.4%)
HCV	0
Other reasons	2 (28.6%)
**Cirrhosis**	5 (71.4%)
**Total bilirubin**	
≥34umol/L	1 (14.3%)
<34umol/L	6 (85.7%)
**Hypoproteinemia**	2 (28.6%)
**Child-Pugh**	
A	4 (57.1%)
B	3 (42.9%)
**PVTT**	
Vp 1	3 (42.9%)
Vp 2	2 (28.6%)
Vp 3	0
**Number of tumors**	
Single	3 (42.9%)
Multiple	4 (57.1%)
**Tumor diameter**	
≥5cm	7 (100%)
<5cm	0

**AFP**	
≥400ng/mL	5　(71.4%)
<400ng/mL	2　(28.6%)
**BCLC**	
A	0
B	2 (28.6%)
C	5 (74.5%)
**Number of cycles of combination therapy**	
2	5 (71.4%)
3	2 (28.6%)
**Complete pathological remission after surgery**	2
**Postoperative recurrence**	1　（12month after surgery）
**Death toll**	0

5 patients used the treatment of FOLFOX-HAIC combined with intilimab and a bevacizumab biosimilar.

**Characteristics**	**Patients**
**Gender**	
Male	5 (100%)
Female	0
**ECOG grade**	
0	3 (60%)
1	2 (40%)
**Etiology**	
HBV	3 (60%)

HCV	1 (20%)
Other reasons	1 (20%)
**Cirrhosis**	1 (20%)
**Total bilirubin**	
≥34umol/L	1 (20%)
<34umol/L	4 (80%)
**Hypoproteinemia**	1 (20%)
**Child-Pugh**	
A	4 (80%)
B	1 (20%)
**PVTT**	
Vp 1	2 (40%)
Vp 2	0
Vp 3	0
**Number of tumors**	
Single	2 (40%)
Multiple	3 (60%)
**Tumor diameter**	
≥5cm	4 (80%)
<5cm	1 (20%)
**AFP**	
≥400ng/mL	3 (60%)
<400ng/mL	2 (40%)
**BCLC**	

A	0
B	4 (80%)
C	1 (20%)
**Number of cycles of combination therapy**	
2	3 (60%)
3	2 (40%)
**Complete pathological remission after surgery**	2
**Postoperative recurrence**	0
**Death toll**	0

1 patient used the treatment of FOLFOX-HAIC combined with lenvatinib and camrelizumab. The patient underwent surgical treatment after 3 cycles of treatment, and pathology showed necrotizing tumor tissue. The patient has no recurrence after surgery and has survived to this day.

Characteristic	
Gender	Male
ECOG grade	1
Etiology	Other reasons
Total bilirubin	<34umol/L
Child-Pugh	A
PVTT	Vp1
Number of tumors	Multiple
Tumor diameter	≥5cm
AFP	≥400ng/mL
BCLC	C
Number of cycles of combination therapy	3
