# Supplementary material for: FOLFOX-HAIC combined with targeted immunotherapy for initially unresectable hepatocellular carcinoma: a real-world study
Source: Front Immunol. 2024 Nov 26;15:1471017. doi: 10.3389/fimmu.2024.1471017 (PMC11628521; doi:10.3389/fimmu.2024.1471017)
Supplement: Supplementary file 2 [file DataSheet2.pdf]

2. The following are some indicators for evaluating changes in liver function during the treatment process. In principle, patients in stage C are not suitable for HAIC treatment. At the time of patient inclusion, stage C patients were excluded. During the treatment process, all patients had baseline CHILD scores in stage A/B, and related liver function abnormalities caused by anti-tumor drugs were controlled after liver protection, keeping them in stage A/B. According to the drug-related liver injury criteria, after two combination treatments, 45 patients were classified as Grade 1 and 6 patients had Grade 2 liver function injury. 49 patients underwent the third combination therapy, with 45 patients in grade 1 and 4 patients with grade 2 liver function injury.

	**Baseline clinical characteristics before treatment (n=51)**	**The first treatment cycle (n=51)**	**The second treatment cycle (n=51)**	**The third treatment cycle (n=49)**
Child-pugh A	29	25	20	20
Child-pugh B	21	26	31	29
ALT⩾5ULN	/	29	36	35
ALP⩾2ULN	/	23	28	26
TBil＜2ULN	/	48	45	45
TBil⩾2ULN	/	3	6	4
Tumor drug-induced liver injury	/	48 patients were classified as Grade 1, and 3 patients had Grade 2 liver function injury.	45 patients were classified as Grade 1, and 6 patients had Grade 2 liver function injury.	45 patients were classified as Grade 1, and 4 patients had Grade 2 liver function injury.
